# Milk Fat Globule Membrane: Structural Organization, Bioactive Constituents, and Therapeutic Applications

**DOI:** 10.3390/foods15091526

**Published:** 2026-04-28

**Authors:** Hongchen Lv, Mengqi Sun, Mengmeng Mi, Shujuan Sun, Yan Zhao, Xinyi Du, Xu Zhang, Mingxia Zhu, Yun Wang, Muhammad Zahoor Khan, Changfa Wang, Mengmeng Li

**Affiliations:** 1 School of Agriculture and Biology, Liaocheng Research Institute of Donkey High-Efficiency Breeding and Ecological Feeding, Liaocheng University, Liaocheng 252000, China; 18963455988@163.com (H.L.); 17862555726@163.com (M.S.); 16652288780@163.com (Y.Z.); duxinyi1289@163.com (X.D.); zhangyifan5353@163.com (X.Z.); zhumingxia@lcu.edu.cn (M.Z.);; 2 Liaocheng Academy of Agricultural Sciences, Liaocheng 252000, China; mi970318@163.com (M.M.); 19861210810@163.com (S.S.); lcwangyun@163.com (Y.W.)

**Keywords:** milk fat globule membrane, bioactive proteins, physiological punctions, separation, application

## Abstract

The milk fat globule membrane (MFGM) wraps around the surface of the milk fat globule, separating the internal lipid core from the external environment. MFGM is a complex trilayer membrane structure composed of polar lipids, sphingolipids, and functional proteins. In recent years, research on the biological characteristics of MFGM has been continuously deepening. It has triggered an exploration of the relationship between MFGM composition, structure, and functional mechanisms. This reveals the potential applications of MFGM in human health and production practices. This review systematically summarizes the composition and structure of MFGM, extraction and preparation techniques, functional mechanisms and the latest research progress in its applications in various fields. This study comprehensively compares the application scope of the MFGM extraction preparation technology. The mechanism of the biological activity of MFGM was further analyzed. Its application value in infant formula, dairy processing, functional foods, drug delivery systems, and cosmetics was evaluated. Nowadays, existing research needs to face numerous challenges, such as some components being unknown and the functional mechanisms not being clear enough. In the future, it is still essential to continuously pay attention to the research progress of MFGM. Further research is needed to accelerate the transformation of MFGM from by-products of dairy processing to multifunctional biomaterials. The purpose is to fully tap its enormous potential in nutrition, health care, and application fields.

## 1. Introduction

The milk fat in breast milk can provide high-efficiency energy and promote the absorption of fat-soluble vitamins. It is an essential nutrient for mammalian infants [1]. Milk fat is mainly composed of triglycerides (TG), which are suspended in mammalian milk in the form of discrete fat droplets of varying sizes [2]. During milk fat globule formation, lipids are synthesized in the endoplasmic reticulum (ER) and bud off. Then, cytoplasmic lipid droplets (CLDs) are formed that are surrounded by a monolayer of phospholipids. Under the mediation of the cytoskeleton, CLDs migrate to the apical membrane and increase in size through fusion. They are secondarily wrapped by the apical membrane. It forms the milk fat globule membrane (MFGM), which has a complex three-layer membrane structure [3] simultaneously accompanied by the formation of cytoplasmic crescents [4]. The MFGM structure is compact and orderly. It contains a single layer of phospholipid membrane inside, and an outer double layer of phospholipid membrane. The space between the two layers of the membrane is rich in functional membrane proteins and polar lipids. They play an important role in the regulation of biological activity [5]. The MFGM performs multiple physiological functions, including involvement in lipid metabolism regulation, promotion of nervous system development, maintenance of intestinal health, and augmentation of immune defense [6]. With the development of MFGM extraction and preparation technology, significant progress has been made in exploring practical MFGM production. It currently shows broad application prospects in the food industry, biomedical field, and cosmetics [7]. However, the composition of MFGM is relatively complex. It is affected by various factors such as the species of mammals, the lactation stage and the processing conditions of dairy products [8,9]. There are still many issues in current MFGM research. Some components have not been clearly identified, the relationship between structure and function is unclear. Modern extraction and purification technologies have not yet formed standardized processes. This restricts the industrial production and application of MFGM [10].

To date, there is a lack of comprehensive reviews addressing MFGM proteins and lipids. This review elaborates on the structural composition, extraction and preparation techniques, physiological functions, and applications in multiple fields of MFGM. It aims to enhance the understanding of the nutritional quality and stability of MFGM and assess its application potential, providing a scientific basis for the efficient development and utilization of MFGM in the future. We used the PubMed and CiteXS search engines. With the keywords of MFGM, bioactive proteins, physiological functions, separation extraction and application fields, a review was conducted by searching the literature from 2002 to 2024. It selected the main bioactive proteins and lipids in MFGM. This summarized their composition and structure, and the extraction and preparation methods. The purpose of this review is to provide an in-depth understanding of the functional mechanisms and application value of MFGM. It also aims to offer valuable insights for both dietary applications and production practices.

## 2. Structural Characteristics and Composition of the MFGM

### 2.1. Structural Features

The internal lipid core of the milk fat globule is rich in TG, and the exterior is wrapped in MFGM. It is rich in bioactive components [11]. The MFGM typically measures 10–20 nm in thickness. It accounts for approximately 2–6% of the total mass of the milk fat globule [12]. During the formation of MFGM, TG is synthesized and accumulated on the cytoplasmic side of the ER in mammalian mammary epithelial cells under the catalysis of lipid synthase. Through the regulation of the Seipin protein, lipid droplets stably form and bud. It forms an initial membrane structure, which subsequently develops into the inner layer of MFGM [13]. Primary lipid microdroplets continuously fuse to form larger CLDs [14]. Cholesterol (CHOL) and sphingolipids in the monolayer membrane of CLDs gradually accumulate. This can provide the material basis necessary for the fusion of CLDs with the apical membrane of mammary epithelial cells [15]. When CLDs reach the apical membrane of mammary epithelial cells, the formation of MFGM is regulated by different pathways [16]. Golgi-derived vesicles continuously transport glycosphingolipids and CHOL to supply the components of the CLD membrane. This provides the necessary material basis for the formation of a complete MFGM [17]. After mature CLDs migrate to the cell membrane, the transmembrane protein butyrophilin (BTN) forms a complex with cytoplasmic xanthine oxidoreductase and the lipid droplet-associated protein adipose differentiation-related protein (ADPH) on the surface of lipid droplets. CLDs are anchored to the apical membrane to participate in the encapsulation process, ultimately forming a complete MFGM structure [18]. Membrane structures originating from the ER and Golgi apparatus continuously envelop fat droplets. They form milk fat globules together with MFGM and secrete them into milk. The MFGM structure conforms to the fluid mosaic model. Lipid molecules form a dynamic matrix, and protein molecules are embedded or cross the lipid matrix [19].

### 2.2. Composition

**Figure 1:**
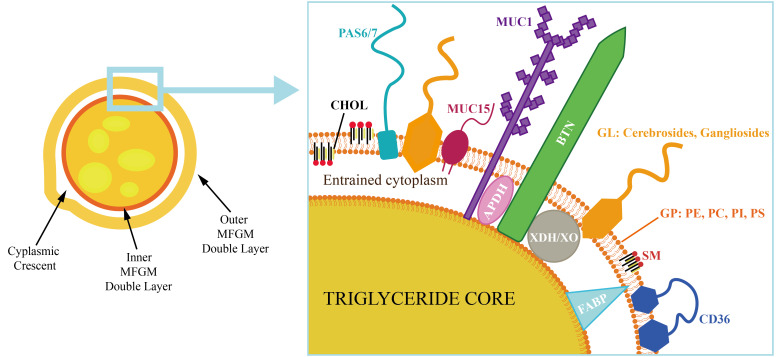
Schematic representation of the three-layer membrane structure of milk fat globules (MFGs) and milk fat globule membrane (MFGM). On the left is the overall structure of the MFG, on the right is a partial schematic diagram of MFGM. The lipids include cholesterol (CHOL), galactolipids (GL), and glycerophospholipids (GP) such as phosphatidylethanolamine (PE), phosphatidylcholine (PC), phosphatidylinositol (PI), phosphatidylserine (PS), and sphingomyelin (SM); proteins include mucins (e.g., mucins, MUC1, MUC15), fatty acid-binding protein (FABP), butyrophilin (BTN), metabolic enzymes (e.g., adipophilin, ADPH, xanthine dehydrogenase/oxidase, XDH/XO), fatty acid transport protein (CD36), and periodic acid-schiff 6/7 proteins (PAS6/7).

The major constituents of the MFGM include proteins, lipids, carbohydrates, and RNA [20]. Proteins and lipids account for more than 90% of its dry weight [21]. The three-layer membrane structure of MFGM from inside to outside is composed of a monolayer of phospholipids, a protein layer, and a phospholipid bilayer [22]. The inner membrane is composed of polar lipids and proteins, directly contacting the internal core. The intermediate layer includes functional enzymes and structural proteins. The outer membrane contains various transmembrane proteins and is covered with glycoproteins (Figure 1).

#### 2.2.1. Milk Fat Globule Membrane (MFGM) Lipids

**Figure 2:**
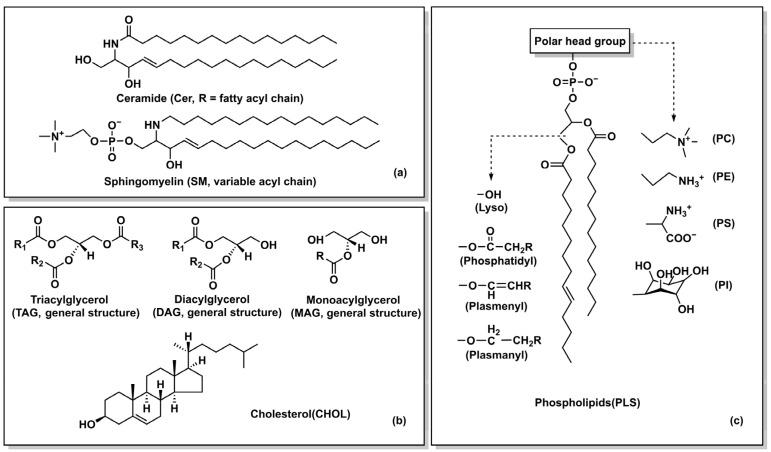
Schematic representation of the chemical structures of different types of MFGM lipids. (**a**) Sphingolipids: Ceramide (Cer), Sphingomyelin (SM). (**b**) Neutral lipids: Triacylglycerol (TAG), Diacylglycerol (DAG), Monoacylglycerol (MAG), Cholesterol (CHOL). (**c**) Phospholipids: The general structure of phospholipids is shown. The left side labels different variants of the backbone, and the right side shows common polar head groups that determine the type of phospholipid, including phosphatidylcholine (PC), phosphatidylethanolamine (PE), phosphatidylserine (PS), and phosphatidylinositol (PI) labeled. The dotted arrows indicate the structural diversity sites of the polar heads of glycerophospholipids. They correspond to the characteristic head groups of major glycerophospholipids, including the head groups of the four types of phospholipids: PC, PE, PS, and PI.

MFGM lipids are predominantly composed of polar lipids, with a minor fraction of neutral lipids [23]. Polar lipids are principally composed of phospholipids and sphingolipids [24]. Glycerophospholipids (GP) constitute 40.6–70% of the total lipid content, encompassing phosphatidylcholine (PC), phosphatidylethanolamine (PE), phosphatidylinositol (PI), and phosphatidylserine (PS) [25]. Within the phospholipid bilayer, GP and sphingomyelin (SM) constitute the structural scaffold. SM and PC are amphipathic and are primarily localized on the outer leaflet of the membrane. PS and PI are mainly located in the inner leaflets of the membrane (Figure 2). The long-chain saturated fatty acids of SM are tightly arranged under the action of Van der Waals forces. The hydrophobic long chains of ceramides intertwine with the fatty acid chains of SM, forming the basis of lipid raft structures through specific interactions [26]. SM has a higher melting point and a more compact arrangement. It helps regulate protein function and maintain membrane structural stability. This plays a key role in signal transduction and transport [27]. The proportion of neutral lipids in MFGM is relatively low, mainly including TG, diglycerides, monoglycerides, and CHOL [28]. Unlike the TG in the core of the milk fat globule, the TGs associated with the MFGM are rich in short-chain fatty acids at the sn-2 position more easily hydrolyzed by gastric lipase, releasing free fatty acids [29]. Diglycerides can regulate blood lipid levels and improve blood sugar control. Monoglycerides have excellent amphiphilicity and are widely used as emulsifiers and moisturizers. CHOL is an essential lipid for the human body and the only precursor for lipoproteins, steroid hormones, and vitamin D [30]. MFGM lipid distribution is asymmetric. They play a key role in maintaining membrane fluidity, regulating lipid raft function, and mediating signal transduction. Table 1 provides an overview of the structure and function of representative lipids in MFGM.

#### 2.2.2. MFGM Proteins

Proteins in the MFGM constitute 25–70% of its dry weight, yet represent only 1–4% of the total protein content in whole milk [40]. Currently known MFGM functional proteins comprise xanthine dehydrogenase/oxidase (XDH/XO), BTN, fatty acid-binding proteins (FABP), periodic acid-Schiff reaction proteins 6/7 (PAS 6/7), cluster of differentiation 36 (CD36), mucins (MUC1, MUC15), ADPH, and others [41]. Table 2 provides an overview of representative proteins in MFGM, the corresponding extraction methods employed, and their functions. The proteins in MFGM are asymmetrically distributed within the membrane. Peripheral membrane proteins are diverse, primarily comprising xanthine oxidoreductase, immunoglobulins and other immune-associated proteins, lipid metabolism-related enzymes, and additional functional proteins originating from the cytoplasm of secretory epithelial cells and granulocytes. They bind non-covalently to the membrane surface. It is easy to dissociate under mild extraction conditions [42]. XDH/XO is located within the membrane and forms a ternary complex with BTN and ADPH. It connects the inner and outer layers of MFGM, maintaining structural integrity. XDH/XO can catalyze the production of reactive oxygen species (ROS) through redox reactions [43]. PAS 6/7 and BTN participate in the regulation of cell proliferation and immune modulation, respectively [44]. Transmembrane transport proteins include CD36 and ATP-binding cassette transporter G2, which mediate fatty acid transport and CHOL efflux, respectively [45]. Structural support proteins include BTN and MUC1, and both form glycosylation modification structures on the membrane surface. They can resist proteolysis and maintain the structural integrity of the gastrointestinal tract [46]. Among enzyme-active proteins, Xanthine Oxidoreductase serves as a key metabolic enzyme mediating ROS regulation.

The binding strength between different proteins and membranes directly affects the separation efficiency and quantitative analysis. There are significant differences in the quantitative analysis of protein in different studies [47]. When studying the function of MFGM protein, we must carefully consider the differences between the source and the experimental methods used in the study.

**Table 2:** Representative proteins in MFGM and their main functions.

Proteins	Isoelectric Point	Molecular Weight (kDa)	Purification Method	Functional Properties	References
Xanthine Oxidase/Xanthine Dehydrogenase (XO, XDH)	7.8	146–150	Ammonium sulfate fractionation and affinity chromatography	Participates in purine metabolism and innate immunity; regulates milk fat globule secretion; maintains structural stability.	[48]
Butyrophilin (BTN)	5.32	66–67	Reverse-phase chromatography	Regulates milk fat globule secretion; activates Vγ9Vδ2 T cells; participates in immune modulation.	[49]
Pregnancy-Associated Serum Proteins 6 and 7 (PAS 6/7)	6.0–6.6	47–59	Ammonium sulfate precipitation and immunoaffinity chromatography	Maintains intestinal epithelial homeostasis; inhibits pathogen infection; regulates gut microbiota balance.	[50]
Cluster of Differentiation 36 (CD36)	<7	76–78	Immunoaffinity chromatography	Mediates long-chain fatty acid transport; regulates inflammation and lipid metabolism.	[51]
Mucin 1 (MUC1)	<4.5	160–200	Lectin affinity chromatography and gel filtration chromatography	Forms epithelial barriers; inhibits pathogen adhesion; serves as target for cancer immunotherapy.	[52]
Mucin 15 (MUC15)	<4.5	95–100	Ion exchange chromatography and gel filtration chromatography	Mediates cell adhesion; maintains MFGM stability; inhibits tumor metastasis.	[52]
Adipophilin (ADPH)	7.5–7.8	52	Ion exchange chromatography	Protects lipid droplets; mediates milk fat secretion; stabilizes MFGM structure.	[53]
Fatty Acid Binding Protein (FABP)	5.0–5.5	13	Ammonium sulfate precipitation, ion exchange chromatography, and gel filtration chromatography	Binds and transports long-chain fatty acids; regulates milk fat synthesis; improves intestinal barrier integrity.	[54]

Abbreviation: XO, XDH: xanthine oxidase/xanthine dehydrogenase; BTN: butyrophilin; PAS 6/7: pregnancy-associated serum proteins 6 and 7; CD36: cluster of differentiation 36; MUC1: mucin 1; MUC15: mucin 15; ADPH: adipophilin; FABP: fatty acid binding protein.

## 3. Extraction and Preparation Techniques of MFGM

### 3.1. Traditional Methods for MFGM Preparation

Centrifugation refers to the use of the density gradient differences in components in the emulsion to separate milk fat globules. It is a classic method for separating MFGM [55]. MFGM isolation usually includes four basic steps: separate milk fat globules from the emulsion, wash the separated milk fat, separate MFGM from the surface of the milk fat globules, and then collect the MFGM fraction [56]. Fat globule membrane separation generally uses centrifugation to enrich milk fat globules from the emulsion. Based on the physical and chemical properties of the emulsion, the parameters of centrifugal force and duration are continuously optimized. This prevents MFGM from prematurely detaching from the surface of fat cells [57].

The washing step is crucial. Under low-temperature conditions of 4–6 °C, milk fat is subjected to 2–4 washes under varying conditions. Water, non-pH-buffered solutions, pH-buffered sucrose solutions, phosphate buffer, or simulated milk ultrafiltrate can be used as washing media. The purpose is to remove contaminating whey proteins and casein [58].

**Figure 3:**
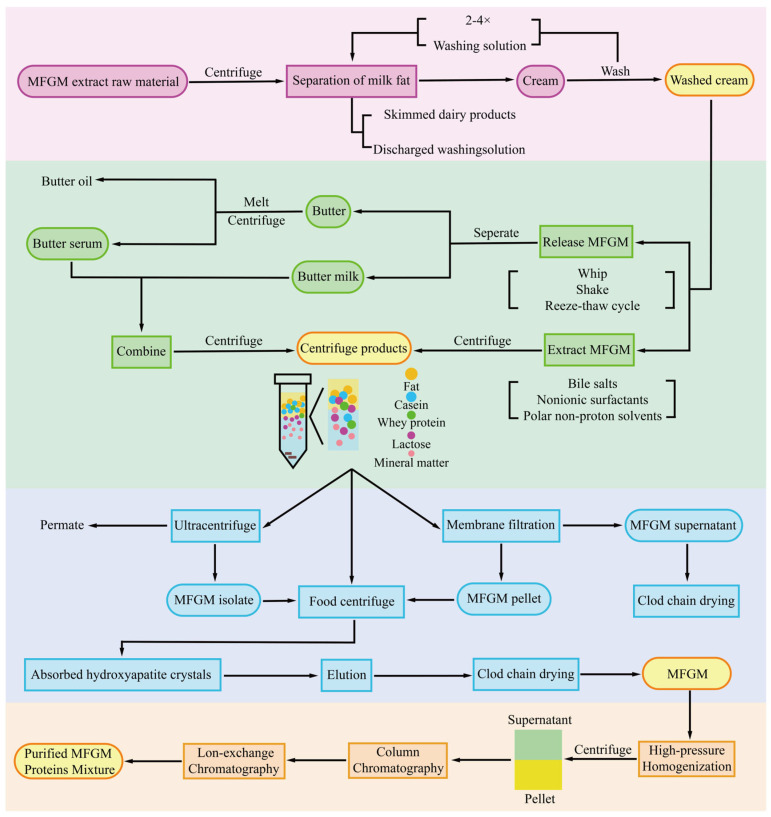
Summary of the MFGM extraction and preparation process flow. The raw materials of MFGM are centrifuged to separate the milk fat and obtain light cream. After 2 to 4 rounds of cleaning, the washed lightweight cream can be obtained. The MFGM can be released through physical methods (such as whipping, oscillation and repeated freezing and thawing), or it can be extracted using reagents such as bile salts and non-ionic surfactants. Centrifugation will yield a product containing fat, casein, and other components. By approaches of ultracentrifugation and membrane filtration, the supernatant and precipitate of MFGM were separated. The precipitate was then subjected to spray drying to obtain MFGM. Further purification using techniques can yield a purified mixture of MFGM proteins.

MFGM is mainly separated from milk fat globules through physical or chemical methods. These methods will destroy the structure of fat globules. This leads to the separation of the membrane from the internal core, promoting the release of MFGM (Figure 3). The collected MFGM concentrate was dried and processed to obtain a final product that is stable, porous and loose in texture. A freeze-drying method is generally used to achieve low-temperature dehydration drying. This can maximize the retention of MFGM’s bioactivity. This method has the drawbacks of high energy consumption and long processing cycles [59].

In traditional methods of preparing MFGM, centrifugation is the most commonly used approach. This method is simple to operate and has low costs, but it has low separation efficiency and low product purity [60]. The heat sterilization method commonly used in traditional processes can cause MFGM proteins to denature and aggregate. This will not only directly damage the integrity of the membrane structure, but also increase the adsorption of casein and whey protein to MFGM. This resulted in a significant decrease in the recovery rate of the MFGM protein in the product [61]. When the target product is MFGM lipids, ultra-high temperature instantaneous sterilization is an important heat treatment step. Although it can cause irreversible conformational changes in certain proteins, the breakdown and restoration of lipids can be promoted.

### 3.2. Modern Industrial Methods for MFGM Preparation

#### 3.2.1. MFGM Isolation

Dairy processing by-products, including buttermilk, butter whey, and cheese whey, serve as the principal raw materials for industrial-scale extraction of MFGM. Butter whey is considered the preferred raw material for the preparation of high-purity MFGM. Cream has emerged as a potential alternative source [62]. While traditional extraction and preparation techniques for MFGM operate under mild conditions, this can preserve the natural structure and biological activity of MFGM. They are limited by insufficient separation efficiency, making them unable to meet modern production demands [63].

With the advancement of technologies, diverse strategies for efficient MFGM extraction from dairy processing by-products have been established. Membrane filtration technology operates under mild conditions. It is amenable to scale-up for production. The separation efficiency is influenced by filtration parameters as well as the physicochemical properties of the source whey. Microfiltration technology separates MFGM particles from small-molecular impurities through microporous sieving. By optimizing temperature and pH, the retention efficiency of MFGM particles and product purity can be significantly improved [64]. Ultrafiltration technology is mainly used for the precise enrichment of MFGM proteins. Selecting the appropriate membrane pore size can enable the effective separation of MFGM proteins from impurities [65].

#### 3.2.2. MFGM Purification

Casein micelles are the main impurities in the MFGM extraction process. They can readily interfere with MFGM separation. Removing casein is a key step in improving MFGM purity [66]. Techniques for casein elimination encompass acid-induced precipitation, chelating salt-based methods, and enzymatic hydrolysis (Figure 3).

The acid precipitation method separates casein from MFGM by adjusting the system pH to the isoelectric point of casein. This causes casein micelles to dissociate and precipitate. If the pH is reduced too far, MFGM proteins may co-precipitate with casein. This results in losses [67]. Acetic acid treatment can lead to partial protein aggregation. Ethylenediaminetetraacetic acid treatment may affect the physicochemical properties of the product. Sodium citrate treatment has relatively low impurity removal efficiency [68,69]. The chelating salt method involves adding sodium citrate or similar agents to break the structure of casein micelles. This can promote their dissociation and reduce the loss of MFGM fragments during dissociation [70]. The enzymatic hydrolysis method uses rennet to control and achieve the separation of casein. Combined with subsequent temperature control and centrifugation, this approach improves the release efficiency of MFGM components. Simultaneously, it can also reduce the damage to the membrane structure. It is currently the preferred method for extracting MFGM for use in infant formula [71].

The hydroxyapatite (HA) adsorption method achieves selective separation of MFGM and casein based on interactions at charged sites [72]. The affinity between MFGM and hydroxyapatite is relatively low. By optimizing parameters such as pH value and ionic strength, the efficient separation of MFGM can be promoted [73]. Table 3 presents a comparison of different MFGM purification methods.

#### 3.2.3. Process Integration

Each single extraction and preparation technique has its shortcomings. They cannot simultaneously meet the demands of industrial production. The integration of multiple technologies represents an important development direction for the extraction and purification of MFGM. The complementary advantages of different technologies can achieve efficient extraction and preparation of MFGM (Figure 3). The integrated technology combining microfiltration and supercritical fluid extraction is continuously maturing. This can enable MFGM to be efficiently purified [74]. Preliminary separation of crude MFGM extract can be achieved through microfiltration pretreatment. This can remove most water-soluble impurities. Subsequently, supercritical fluid extraction technology is used to remove neutral lipids from the crude extract. This effectively avoids damage to the bioactive components of MFGM caused by heat treatment and chemical solvents [55]. Enzymatic pretreatment integrated with membrane filtration uses cream as the raw material. By controlling the maturation and demulsification conditions, buttermilk is obtained. Rennet is added for the coagulation reaction, which loosens the casein floc structure. It allows the MFGM components to be fully released into the buttermilk serum. Membrane filtration is then used to enrich the MFGM. Ultimately, the efficient simultaneous recovery of MFGM protein and phospholipids was achieved [75]. Microfluidic separation technology precisely separates MFGM by utilizing biomolecular binding sites and fluid barrier effects. This maximally maintains the natural three-layer membrane structure of MFGM [76]. To obtain the protein mixture in MFGM, homogenization is the most common mechanical treatment used in the processing stage. Under high-pressure homogenization conditions, the arrangement of membrane proteins and phospholipids in the MFGM changes. Some of the original proteins detach from the interface. Exogenous proteins can subsequently re-adsorb at the interface. This can affect the interfacial thermal stability of milk fat globules and promotes the recovery of MFGM proteins [77].

## 4. Physiological Functions and Mechanisms of MFGM

### 4.1. Regulation of Lipid Metabolism

**Figure 4:**
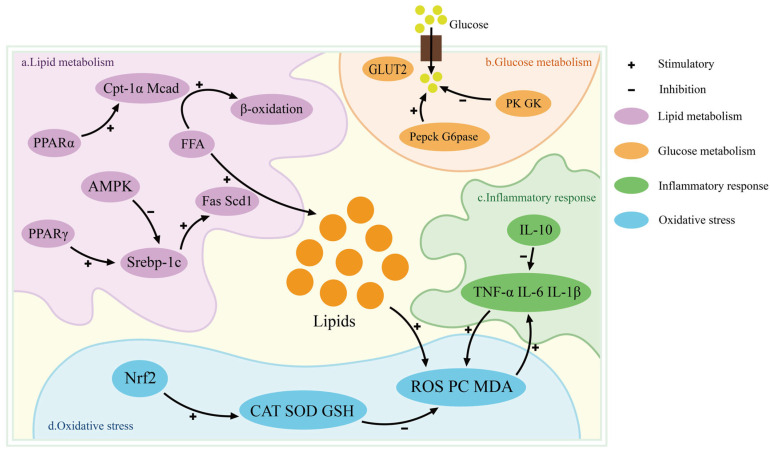
Regulation of lipid metabolism by MFGM. The figure depicts the regulatory network encompassing lipid metabolism, glucose metabolism, inflammatory responses, and oxidative stress. The arrows represent the interactive regulatory relationship among lipid metabolism, oxidative stress, glucose metabolism and inflammatory response. (**a**) Lipid Metabolism: PPARα: peroxisome proliferator-activated receptor alpha; PPARγ: peroxisome proliferator-activated receptor gamma; AMPK: AMP-activated protein kinase; Cpt-1α carnitine palmitoyltransferase-1α; Mcad: medium-chain acyl-CoA dehydrogenase; Fas: fatty acid synthase; Scd1: stearoyl-CoA desaturase 1; FFA: free fatty acid; Srebp-1c: sterol regulatory element-binding protein-1c. (**b**) Glucose Metabolism: GLUT2: glucose transporter type 2; Pepck: phosphoenolpyruvate carboxykinase; G6Pase: glucose-6-phosphatase; PK: pyruvate kinase; GK: glucokinase. (**c**) Inflammatory Responses: TNF-α: tumor necrosis factor-α; IL-6: interleukin-6; IL-1β: interleukin-1β; IL-10: interleukin-10. (**d**) Oxidative Stress: Nrf2: nuclear factor erythroid 2–related factor 2; CAT: catalase; SOD: superoxide dismutase; GSH: glutathione; PC: protein carbonyls; ROS: reactive oxygen species; MDA: malondialdehyde.

MFGM plays a critical role in lipid metabolism regulation. This is primarily achieved by inhibiting lipid accumulation and enhancing glucose homeostasis. It can also work synergistically to alleviate oxidative stress and regulate inflammatory responses (Figure 4). The role of MFGM in inhibiting lipid accumulation is mainly reflected in two aspects: inhibiting synthesis and promoting degradation. MFGM intervention markedly downregulates the expression of key hepatic lipogenic genes, including fatty acid synthase, sterol regulatory element-binding protein-1c (SREBP-1c), and stearoyl-CoA desaturase 1. Simultaneously, it upregulated the expression of genes involved in fat breakdown and β-oxidation [78]. The activity regulation of SREBP-1c plays a key role in MFGM inhibition of lipid synthesis. In its inactive state, SREBP-1c binds to the SREBP cleavage-activating protein (SCAP) on the ER membrane through its transmembrane domain. This can form a stable complex [79]. Upon depletion of intracellular sterol levels, the SCAP-SREBP complex is translocated from the ER to the Golgi apparatus. It undergoes sequential cleavage by site-1 protease and site-2 protease. This proteolytic processing releases the N-terminal transcriptional domain of SREBP-1c. This can enable its translocation into the nucleus and subsequent activation of lipogenic gene expression. MFGM can inhibit the Golgi transport of this complex to reduce the nuclear translocation of Srebp-1c. The signal transduction of lipid synthesis is blocked [80]. MFGM facilitates lipid catabolism primarily through activation of the peroxisome proliferator-activated receptor alpha (PPARα) signaling pathway [81]. MFGM can significantly upregulate the expression of PPAR-α downstream target genes carnitine palmitoyltransferase 1α and medium-chain acyl-CoA dehydrogenase. This will accelerate the mitochondrial fatty acid β-oxidation process [82,83]. MFGM further enhances the transcriptional activity of PPAR-α through phosphorylation of AMP-activated protein kinase (AMPK). It can regulate lipid metabolism by inhibiting SREBP-1c activity through AMPK-mediated phosphorylation [84]. With respect to glucose metabolism, MFGM reduces hepatic endogenous glucose production by downregulating the expression of key gluconeogenic genes phosphoenolpyruvate carboxykinase and glucose-6-phosphatase. Simultaneously, it upregulates glycolysis-related genes encoding pyruvate kinase and glucokinase. This will accelerate glucose breakdown and utilization to maintain blood glucose homeostasis [85]. MFGM further modulates gut microbiota metabolism, leading to the production of short-chain fatty acids. They can activate intestinal L-cells to secrete glucagon-like peptide-1. It can enhance insulin secretion and inhibits the release of glucagon [86]. In the process of oxidative stress and inflammation regulation, MFGM activates the nuclear factor erythroid 2-related factor 2 (Nrf2) pathway. This can promote the expression of antioxidant enzymes including superoxide dismutase, catalase and glutathione peroxidase. It can reduce the accumulation of ROS and malondialdehyde. The reduction of these oxidative by-products can inhibit the release of pro-inflammatory cytokines and upregulate the expression of the anti-inflammatory cytokine. This can alleviate metabolism-related inflammation [87].

### 4.2. Gut Health

**Figure 5:**
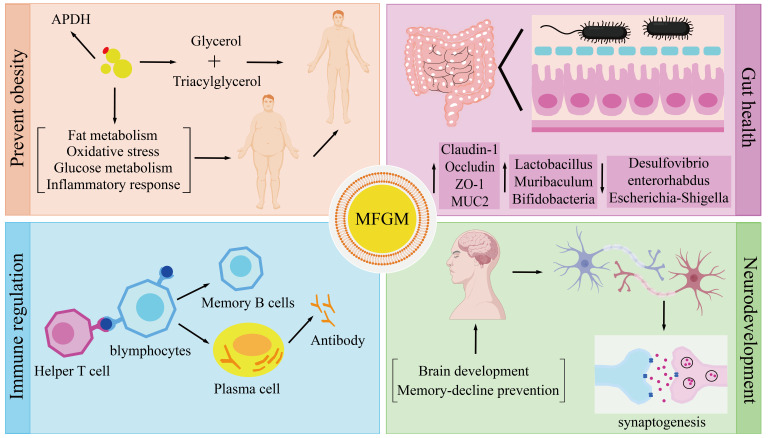
Schematic illustration of the major biological functions and underlying mechanisms of MFGM. Regarding lipid metabolism, MFGM can prevent obesity by regulating lipid metabolism, reducing oxidative stress, improving glucose metabolism and inhibiting inflammatory reaction. The arrow indicates the regulatory pathway through which MFGM exerts its anti-obesity effect. In terms of intestinal health, MFGM improves intestinal health by up-regulating tight junction-associated proteins and intestinal microbiota composition. The upward arrow indicates the increase in the expression and abundance of intestinal tight junction proteins, mucins, and beneficial gut symbiotic bacteria. The downward arrow indicates the reduction in the abundance of intestinal opportunistic pathogens and harmful bacteria. In terms of immune regulation, MFGM also plays a role in immune regulation through a variety of ways. The arrow indicates the progressive process of the adaptive immune response regulated by MFGM. In the aspect of neurodevelopment, MFGM participates in the process of neurodevelopment by supporting brain development, promoting synapse formation and preventing memory decline. The arrow indicates the regulatory pathway of MFGM’s role in promoting neural development. (Created with BioGDP.com [105]).

The phospholipid components in MFGM can integrate into the intestinal epithelial cell membrane, supplementing the mucosal lipid layer. It can promote the formation of lipid raft structures, maintaining normal membrane fluidity and signaling functions (Figure 5). MFGM can activate signaling cascades to enhance both the expression and membrane localization of tight junction proteins. The purpose is to reduce intestinal permeability [88]. The mucin components in MFGM can promote the secretion of intestinal mucin 2 (MUC2) and mucin 4 (MUC4) and the proliferation of goblet cells, as well as thicken the mucus layer to provide a barrier function for resisting the invasion of pathogens such as *Helicobacter pylori* [89]. Antimicrobial proteins exist in MFGM. They act synergistically with phospholipids to exert potent antimicrobial effects. Glycoproteins inhibit pathogen adhesion and invasion.

Certain MFGM components that resist host digestion can function as prebiotics, stimulating the proliferation of bifidobacteria and lactobacilli. When combined with probiotics, it enhances the survival and adhesion capabilities of beneficial bacteria in the intestinal tract [90]. The glycosphingolipid substances are produced by MFGM metabolism. They can acidify the intestinal lumen. This will inhibit the colonization of pathogenic bacteria such as Enterococcus in the intestines. It alleviates the inflammation caused by endotoxins [91]. Studies show that infant formula containing MFGM can render the intestinal barrier function of infants comparable to that of breastfed babies. It may reduce diarrhea [92].

### 4.3. Immune Regulation

The bioactive components in MFGM play a central role in maintaining immune homeostasis in the host [93]. MFGM targets the regulation of innate immune cell functions and directly combats pathogens. This constitutes the first line of defense in the immune system [94]. At the level of immune cell activation, macrophages are regulated by MFGM. phospholipids activate pattern recognition receptors on the surface of macrophages. They enhance their capacity to phagocytose and remove apoptotic epithelial cells. This can downregulate the secretion of pro-inflammatory cytokines such as tumor necrosis factor-α (TNF-α) and interleukin-6 (IL-6). This will reduce tissue damage caused by excessive neutrophil activity [95]. In antiviral defense, glycoproteins are abundant in MFGM. They can use surface sialic acid glycan structures to mimic host cell receptors. Specifically, it can bind to viral envelope proteins. The attachment and invasion of the virus are blocked [96].

MFGM plays a critical role in the fine regulation of adaptive immunity by modulating T cell subset differentiation and B cell function. It provides sustained immune protection and maintains immune tolerance (Figure 5). In T cell homeostasis regulation, the activation of dendritic cells mediated by MFGM can specifically induce the differentiation of regulatory T cells. It can secrete anti-inflammatory mediators to inhibit excessive immune responses [97]. In terms of B cell function regulation, the immunoglobulins present in MFGM can directly participate in humoral immunity. The phospholipid components can promote the differentiation of B cells into plasma cells and can stimulate the production of specific antibodies [98].

### 4.4. Neurodevelopment

The SM and its metabolic product ceramide, which are abundant in MFGM, can promote neurological development in infants and young children [99]. SM can directly participate in the formation and maintenance of myelin to provide a structural basis for efficient nerve signal transmission [100]. The SM metabolite ceramide can regulate neuronal function in multiple ways. In early development, ceramide participates in the establishment of neuronal polarity and the directional extension of axons. This lays the foundation for the construction of neural circuits [101]. Following the formation of neuronal connections, ceramide activates critical signaling pathways to upregulate expression of postsynaptic density protein 95. This effect synergizes with activation of the extracellular regulated protein kinases 1/2 signaling pathway. This promotes the coordinated expression of synapsin family proteins to enhance synaptic plasticity and transmission efficiency [102]. Collectively, these mechanisms constitute the central network. Squalane supports the proper development and functional maturation of the nervous system through the central network (Figure 5) [103]. Glycosylated ceramides, characterized by abundant oligosaccharide chains, markedly increase blood–brain barrier permeability by specifically interacting with the glycocalyx of brain endothelial cells. This facilitates myelin development and repair [104].

## 5. Applications of MFGM in the Food Industry

### 5.1. Infant Formula

**Figure 6:**
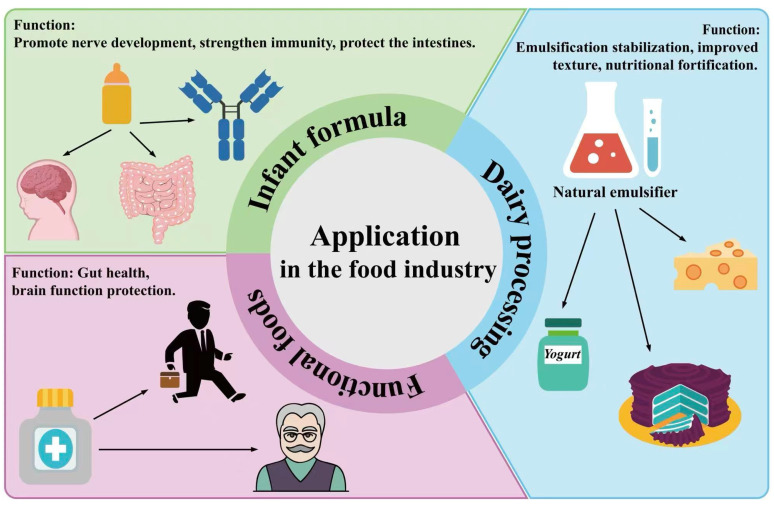
Applications of MFGM in the food industry. In infant formula, it supports neurodevelopment and intestinal health and enhances immune function. In the field of dairy products processing, as a natural emulsifier, it is widely used in cheese, yogurt, dessert and other products. Functional foods can be customized for specific groups to support intestinal health and protect cognitive function. (Created with BioGDP.com [105]).

MFGM serves as the natural bioactive membrane structure that surrounds fat globules in breast milk. It is widely used in the upgrading of infant formula to more closely mimic breast milk (Figure 6). The composition and structure of MFGM are similar to the bioactive nutritional components of breast milk. This provides infants with key precursor substances needed for neurodevelopment, immune system building, and gut maturation without additional modification [106]. MFGM ingredients exhibit sufficient stability to withstand the high-temperature sterilization and spray-drying processes employed in infant formula production. Studies have shown that the growth parameters of infants supplemented with MFGM are not significantly different from those of breastfed or standard formula-fed groups [107]. With respect to physiological functions, MFGM exerts influence in three major domains of early-life development. By promoting myelination, MFGM enhances the efficiency of neural signal transmission [108]. Concurrently, it can regulate the intestinal microbiota structure to maintain intestinal health and strengthen immune regulatory functions [109].

In industrial applications, MFGM is primarily incorporated into infant formulas using three approaches: standardized raw material supplementation, synergistic formulation, and phased adaptation strategies. In practice, MFGM-enriched whey protein concentrate is commonly used as the delivery matrix. The supplementation can be adjusted according to the phospholipid content to meet the physiological needs of the infant [110]. It can be further aligned with the nutritional profile of breast milk by combining it with other ingredients [111]. Stage-specific infant formulas have been developed to cater to the developmental requirements of infants at distinct growth stages. Nutritional requirements can be met by adjusting the purity of MFGM, the phospholipid ratio, and the composition of composite ingredients [112].

### 5.2. Dairy Product Processing

MFGM, as a natural emulsifier, demonstrates considerable application value in the processing of fermented dairy products, cheese, and cream products. It is becoming a key ingredient for enhancing product quality and nutritional value (Figure 6) [113]. In the field of fermented dairy products, MFGM is often added in the form of an extract to the raw milk of yogurt or fermented milk beverages at a concentration of 0.1–0.5% of the total mass. This can effectively inhibit whey separation during fermentation and storage. It will improve product viscosity and texture uniformity to extend the shelf life [114,115]. In comparison with conventional emulsifiers, the synergistic effect of MFGM and lactic acid bacteria metabolism can also optimize the product’s flavor profile and improve product quality. This can achieve nutritional enhancement [116].

Beyond fermented dairy products, MFGM exhibits substantial application potential in the processing of cheese and cream-based products. Primarily derived from buttermilk, MFGM is commonly incorporated in the form of buttermilk powder as a natural functional modifier. In low-fat cheese, it can replace a proportion of the fat. This significantly enhances the physical properties of the product and ameliorates issues of rough texture and easy crumbling in low-fat cheese [117]. In cream-based dairy products, MFGM can inhibit fat separation and crystallization to establish a stable interfacial layer. It ensures the uniformity of texture and spreadability of the product during its shelf life [118].

### 5.3. Functional Foods

MFGM is a natural composite ingredient rich in bioactive components. It is widely used in nutritional supplements specifically for the elderly and office workers. This mainly targets brain function protection and intestinal health maintenance (Figure 6) [119]. For the problem of cognitive decline and decreased motor coordination in the elderly, the phospholipids in MFGM exert protective effects by promoting neural plasticity and inhibiting neuroinflammation. This will help to maintain memory, improve neuromuscular junction function and delay muscle atrophy [120,121]. In office workers chronically subjected to high stress and sustained cognitive demands, MFGM modulates the hypothalamic–pituitary–adrenal (HPA) axis to alleviate chronic stress and mental fatigue. This concurrently enhances subjective sleep quality. It will provide nutritional support that contributes to sustained cognitive performance and work efficiency [122]. Regarding gut health maintenance, MFGM improves intestinal barrier integrity and modulates gut microbiota composition. This attenuates intestinal inflammation and reduces oxidative stress. It can actively mitigate intestinal aging-related damage in the elderly and intestinal functional disorders caused by irregular lifestyles in office workers [123]. MFGM has excellent processing stability and biocompatibility. This can be widely incorporated into various product forms. It can be combined with ingredients like probiotics and prebiotics to meet the needs of both population groups [124].

## 6. Potential Applications of MFGM

### 6.1. Applications of MFGM in Medicine

Phospholipids derived from MFGM can form spherical liposome structures owing to their amphiphilicity. The molecular head carries a hydrophilic phosphate group, while the tail carries a hydrophobic fatty acid chain [125]. This encapsulation advantage stems from the specific composition of MFGM phospholipids. Compared to the slightly larger diameter of soybean lecithin liposomes, MFGM-derived liposomes further enhance the encapsulation of hydrophilic substances [126]. This encapsulation advantage stems from the specific composition of MFGM phospholipids. Compared to the slightly larger diameter of soybean lecithin liposomes, it further enhances the encapsulation of hydrophilic substances [127]. By utilizing a meticulous cream concentration process, the MFGM component rich in 7–8% phospholipids can be obtained. This can further enhance the encapsulation performance of the liposomes and the reproducibility between batches [128].

The ‘lipid rafts’ in MFGM can enhance the rigidity and degradation resistance of liposome membranes to improve their stability. It effectively resists enzymatic hydrolysis by lipases in the gastrointestinal tract and protein adsorption and aggregation in the blood [129]. Glycoproteins on the surface of liposomes interact specifically with sialic acid receptors expressed by intestinal epithelial cells, which can mediate targeted drug delivery. Gangliosides enriched in MFGM synergistically promote brain-targeted drug delivery by facilitating the activity of the CD36 transporter [130]. By integrating enzyme-responsive release mechanisms, liposomes can achieve precise drug delivery to lesion sites. This effectively overcomes clinical challenges associated with the poor solubility and low targeting efficiency of hydrophobic drugs [131].

### 6.2. Applications of MFGM in Cosmetics

The natural phospholipid–protein complex structure of MFGM is enriched with bioactive components. It exhibits unique potential for enhancing skin barrier repair [132]. SM in MFGM is a key precursor of ceramides in the stratum corneum of the skin. It aligns with the skin lipid metabolism pathway. This can directly integrate into the lipid bilayer of the stratum corneum. It will show significantly better barrier integrity and skin compatibility than chemically synthesized ceramides [133]. MFGM is enriched with SM predominantly containing long-chain fatty acids. Ceramides produced via SM hydrolysis can specifically reinforce the stability of the long-period lamellar structure within the stratum corneum. MFGM products that meet cosmetic raw material standards can achieve phospholipid enrichment through concentrated cream refining processes. This can meet the production requirements of cosmetics [134]. PC in MFGM represents a pivotal bioactive component for modulating skin hydration. It exerts its effects that enhance both cutaneous moisture retention and barrier integrity. PC not only forms a hydrophobic moisturizing layer by interacting with corneocyte-associated mucins to reduce transepidermal water loss, but also stimulates the synthesis and release of endogenous natural moisturizing factors within keratinocytes [135]. Gangliosides serve as characteristic components of MFGM. They can ameliorate discomforts such as dryness and itching caused by damage to skin nerve endings by regulating neural signal transmission. This makes them ideal ingredients for the development of multifunctional cosmetics [136]. Skincare products based on MFGM have already been preliminarily applied in the adjuvant care of dry skin diseases [137].

## 7. Conclusions and Future Perspectives

The milk fat globule membrane represents a versatile, naturally occurring biomaterial with demonstrated structural, nutritional, and bioactive properties. This review has summarized the research progress in the structure, extraction, preparation, functional mechanisms, and multi-field applications of MFGM. It provides a framework for understanding the industrial production and application of MFGM.

This field has developed from the centrifugal recovery of raw materials to the industrial processing of membrane technology and integrated process. It has established applications in infant nutrition, functional foods, pharmaceutical delivery systems, and cosmeceuticals. The advancement of technology has enabled a continuous exploration of the biological properties of the components of MFGM. Nonetheless, it is urgent to address the major challenges that remain to fully realize the practical value of MFGM. Changes in components caused by biological and technical factors require standardized quality indicators. Bioactive constituents have been continuously identified, but the functional interrelationships between them are still not completely clarified. The lack of uniform criteria in extraction and preparation methods and quality standards further limits research progress.

In conclusion, MFGM has transitioned from a dairy processing by-product to a scientifically validated functional biomaterial with demonstrated nutritional and therapeutic applications. In the future, it is necessary to address the current knowledge gaps through research, clinical validation and technological innovation. This will allow the full potential of MFGM to be realized as a component of precision nutrition, targeted therapy, and preventive health strategies. The convergence of processing innovation and clinical application positions MFGM at the forefront of next-generation bioactive ingredients. This can address critical needs in pediatric nutrition, metabolic health, cognitive function, and immune resilience across the lifespan.

## Figures and Tables

**Table 1:** Structural and functional characteristics of representative lipids in MFGM.

Lipids	Structural Characteristics	Functional Properties	References
PC	Glycerol backbone with choline headgroup linking two fatty acid chains; amphipathic molecule.	Maintains membrane stability; promotes lipid emulsification and absorption; provides choline to support neurodevelopment.	[31]
PE	Glycerol backbone with ethanolamine headgroup; capable of forming intermolecular hydrogen bonds.	Anchors membrane proteins; regulates signal transduction; enhances intestinal mucosal barrier function.	[32]
PI	Glycerol backbone with polyhydroxy inositol headgroup; capable of phosphorylation modifications.	Mediates signaling pathways; regulates membrane fluidity; participates in immune activation.	[33]
PS	Glycerol backbone with serine headgroup; negatively charged; enriched on the inner leaflet of the membrane.	Promotes synaptogenesis; facilitates clearance of apoptotic cells; inhibits inflammation.	[34]
SM	Sphingosine backbone with phosphocholine headgroup; contains long-chain hydrophobic tails.	Forms lipid rafts; promotes myelin development; inhibits pathogen adhesion.	[35]
Cerebrosides	Ceramide linked to monosaccharides via β-glycosidic bonds.	Participates in cell recognition; enhances intestinal barrier function; regulates gut microbiota balance.	[36]
Gangliosides	Composed of hydrophobic ceramide and hydrophilic oligosaccharide chains; negatively charged.	Promotes cognitive development; prevents pathogen infection; regulates immune responses.	[37]
TG	One glycerol molecule esterified with three fatty acid molecules; enriched in medium-chain fatty acids at the sn-2 position.	Provides rapid energy supply; easily digested and absorbed.	[38]
CHOL	Steroidal tetracyclic structure containing hydroxyl and isooctyl side chains.	Regulates membrane fluidity; serves as precursor for vitamin D3 and steroid hormones; promotes lipid absorption.	[39]

Abbreviation: PC: phosphatidylcholine; PE: phosphatidylethanolamine; PI: phosphatidylinositol; PS: phosphatidylserine; SM: sphingomyelin; TG: triglyceride; CHOL: cholesterol.

**Table 3:** Comparison of MFGM purification Methods.

Purification Method	Affinity or Adsorption Rate	Advantages	Disadvantages
Acid precipitation method	90–99%	Easy to operate, low cost, suitable for large-scale processing	The natural component properties of whey protein are easily destroyed; the removal rate is limited.
Chelate salt method	70–90%	Mild operating conditions	High cost; safety hazards
Enzymatic hydrolysis	80–95%	Specific and mild under certain conditions	Higher cost; affects product flavor
Hydroxyapatite adsorption method	80–95%	Selective affinity; strong adsorption capacity	High cost; conditions are difficult to control

## Data Availability

No new data were created or analyzed in this study. Data sharing is not applicable to this article.
